# STING regulates NETs formation by activating GSDMD in influenza viral pneumonia

**DOI:** 10.3389/fimmu.2025.1598902

**Published:** 2025-07-01

**Authors:** Rongrong Huang, Ranran Chen, Lijuan Xing, Lianhao Wu, Wenwen Zhu, Junsong Jing, Ting Zhou, Yueguo Wu, Sheng Zhang, Zhenqiang You

**Affiliations:** ^1^ School of Basic Medical Sciences & Forensic Medicine, Hangzhou Medical College, Hangzhou, China; ^2^ School of Public Health, Hangzhou Medical College, Hangzhou, China; ^3^ Key Discipline of Zhejiang Province in Public Health and Preventive Medicine (First Class, Category A), Hangzhou Medical College, Hangzhou, China; ^4^ Center for Safety Evaluation and Research, Hangzhou Medical College, Hangzhou, China

**Keywords:** viral pneumonia, STING, NETs, GSDMD, anti-inflammatory

## Abstract

**Background:**

Viral pneumonia is the most common and lethal pandemic disease, but there are no broad-spectrum antiviral drugs with high genetic barriers to resistance. To elucidate the mechanisms of viral pneumonia progression and potential targets for its treatment.

**Methods:**

Viral pneumonia models were induced by the PR8 virus strain in wild-type (WT) and STING knockout (STING-KO) mice. Series of molecular biology techniques were used to evaluate the severity of pneumonia and cytokine levels.

**Results:**

In this study, STING (stimulator of interferon genes) was activated in the lungs of virus-infected mice, leading to cytokine production and amplification of the immune response, thereby causing rapid deterioration of symptoms. Furthermore, excessive activation of innate immune response via STING was prevented by a STING inhibitor (C-176), which significantly reduced viral lung inflammation. The formation of neutrophil extracellular traps (NETs) was similarly suppressed during viral pneumonia treatment with STING inhibitors (C-176), and NETs formation and STING expression were positively correlated, indicating that STING plays an important role in NETs formation. Symptoms of pneumonia in STING-KO mice infected with PR8 were significantly milder than those in WT mice, and NETs were less likely to form in the lung tissue of STING-KO mice. Additionally, transcriptomic analysis revealed that STING-mediated regulation of NETs may be associated with gasdermin D (GSDMD), and immunoprecipitation experiments revealed that STING, GSDMD, and NETs-related proteins interact with each other. Immunofluorescence assays revealed that in neutrophils from WT mice, STING and GSDMD were colocalized on the membrane after viral infection, whereas in neutrophils from STING-KO mice, GSDMD expression was decreased after exposure to the virus.

**Conclusions:**

Our study demonstrated that targeted intervention with STING alleviated pneumonia by inhibiting inflammation and NETs formation. The study also revealed that blocking STING could inhibit the activation of GSDMD to inhibit NETs formation, slowing the progression of viral pneumonia.

## Introduction

Viral pneumonia is an inflammatory disease caused by viral invasion of the respiratory system and lungs (1). After infection of the upper respiratory tract, viruses replicate within bronchial, bronchiolar, and alveolar epithelial cells, leading to the rupture and necrosis of these cells. This process results in the leakage of tissue fluid and attracts neutrophils, monocytes, and natural killer cells to the site of infection through chemotaxis. Consequently, capillary damage is accompanied by the release of numerous inflammatory factors and reactive oxygen species. The resulting cytokine storm provokes severe inflammation in the lung tissue, culminating in extensive tissue damage, blood vessel permeability, and the development of pulmonary edema. In patients, this cascade of events leads to hypoxia, potentially progressing to acute respiratory distress syndrome, shock, and other severe complications (2–4). Influenza viruses undergo rapid and robust mutation, which can cause pandemics of viral lung inflammation, and have caused an unparalleled health, social, and financial crisis. Moreover, the clinical treatment options currently available for influenza virus infections are limited to supportive care, which focuses on inhibiting viral replication, relieving symptoms, suppressing the body’s excessive inflammatory response, regulating immune homeostasis, and protecting organs (5). Antiviral medications such as neuraminidase inhibitors and viral polymerase inhibitors shorten the duration of influenza symptoms, limiting virus spread and lowering the risk of death from influenza (6). However, viruses exhibit typical genetic diversity; thus, the treatment of viral pneumonia remains challenging (7). Hence, further investigation is necessary to clarify the intricate relationships among the influenza virus, the host immune reaction, and inflammatory mechanisms to develop more effective strategies to prevent and treat influenza virus-induced inflammation and complications.

Biological barriers and the innate immune system constitute the initial line of defense against pathogen invasion, which is critical for antiviral immunity (8). STING, an essential protein at the junction of the innate immune signaling pathway that was initially identified within the context of cancer–immunity interplay, is a pivotal mediator in the detection of foreign entities derived from pathogens such as viruses and bacteria, along with endogenous molecules indicative of self-harm within the organism (9). The STING pathway plays a crucial role in orchestrating cellular immune responses to intracellular abnormalities or exogenous DNA fragments. The pathway triggers the activation of key molecules such as TANK-binding kinase 1 (TBK1), interferon regulatory factor 3 (IRF3), and nuclear factor kappa-B (NF-κB), thereby stimulating immune responses that include the generation of type I interferons (IFNs), which have antiviral effects and help to prevent additional lung injury (10–12). However, prolonged activation of the STING pathway results in increased production of type I IFNs and inflammatory cytokines, initiating an inflammatory response. Accumulating evidence indicates that overactivation of the STING pathway is involved in the pathogenesis of a wide range of disorders and diverse pathological processes, including cell death, tissue damage and autoimmune diseases (13). Studies in mouse models have indicated that the manifestation of autoimmune symptoms and myocarditis due to an overabundance of type I IFNs may be averted through the disruption of STING gene expression or the suppression of STING activity (14). These findings underscore the essential requirement for negative modulation of STING signaling cascades, with evidence indicating the efficacy of STING inhibition as a therapeutic approach for managing inflammatory conditions (15, 16).

Excessive lung inflammation brought on by influenza virus infection is characterized by the buildup of inflammatory cytokines and the destruction of lung tissue, which is fatal in both human patients and animal models (17–19). Innate immune cells, especially neutrophils and macrophages, can be activated by pathogenic molecules in the blood during viral pneumonia, which can lead to their degranulation and phagocytosis, ultimately causing systemic inflammation (20). Since NETs were first reported by Brinkman in 2004, they have been found to be involved in the pathophysiologic processes of pulmonary disease, such as acute lung damage (21). Extracellular double-stranded DNA, myeloperoxidase (MPO), neutrophil elastase, cathepsin, and histones compose the extracellular meshwork structures known as NETs, which are secreted by neutrophils (22). To capture and eliminate pathogens and combat illness, NETs sustainably release chromatin and antimicrobial proteins (23). However, it is generally agreed that NET formation is a double-edged sword of innate immunity (24). Excessive formation of NETs has recently been identified to be a novel mechanism, contributing to exacerbated inflammation, the onset of autoimmune diseases, and thrombus formation (25–27). The inhibition of further NETs formation was shown to lessen lung damage and the inflammatory response in sepsis-associated acute pulmonary injury and acute respiratory distress syndrome (28–30). The intracellular signaling mechanisms of NETs and distinctly resemble those of STING, as has been elucidated under various inflammatory conditions. However, the interconnection among NETs, STING, and viral pneumonia resulting from influenza virus infection remains inadequately supported by current evidence.

Among the gasdermin family members, GSDMD has attracted considerable attention due to its role as the primary executor of cellular pyroptosis. Acting downstream of inflammatory caspases, GSDMD is pivotal for regulating cellular inflammatory responses (31). During inflammasome-induced pyroptosis, GSDMD undergoes hydrolytic cleavage by activated caspase-1, generating an active fragment capable of forming membrane pores. This process facilitates the release of IL-1β and IL-18 through unconventional protein secretion (32). Notably, noncanonical inflammasome pathways activate human Caspase-4/5 and mouse Caspase-11, leading to the formation of GSDMD pores that release potassium, triggering NLRP3 inflammasome activation and subsequent maturation of IL-1β/IL-18. The resulting GSDMD pores induce cell swelling and release mature cytokines (33, 34). GSDMD is expressed primarily in immune cells such as macrophages and monocytes. GSDMD-mediated lysis is promoted by classical inflammasomes in neutrophils, but the absence of GSDMD weakens IL-1β secretion from neutrophils. Neutrophil elastase-mediated GSDMD cleavage is a crucial event in phorbol 12-myristate 13-acetate (PMA) induced NETosis (35), suggesting that GSDMD may facilitate neutrophil membrane permeabilization and NETs formation.

In this study, we used the PR8 virus to establish mouse models of viral pneumonia and systematically investigated its pathogenesis, focusing on the role of the STING pathway. We further investigated the interaction between the STING pathway and the formation of NETs in PR8-induced viral pneumonia, evaluating the therapeutic potential and underlying mechanisms of STING-targeted interventions.

## Materials and methods

### Preparation and administration of drugs

The A/H1N1/Puerto Rico/8/1934(PR8) mouse-adapted strain of influenza virus was provided by Zhejiang University (Hangzhou, China). The virus was stored in equal portions at -80°C. The influenza virus was diluted in a sterilized physiological saline solution (SLYS-001, KERONG, Guangzhou, China) until further use. C-176 was purchased from Med-Chem Express (Princeton, NJ, USA). Corn oil was purchased from Machlin Co., Ltd. (Shanghai, China). Dimethyl sulfoxide (DMSO) was purchased from Aladdin Co., Ltd. (Shanghai, China). The following antibodies were used: anti-NF-κB (#8242T) 1:1000, anti-p-NF-κB (#3039S) 1:4000, anti-IRF3 (#11904T) 1:1000, anti-p-IRF3 (#29047S) 1:5000, anti-p-STING (#72971s) 1:2000, anti-TBK1 (#38066s) 1:1000, anti-p-TBK1 (#5483T) 1:5000, anti-GAPDH (#14C10) 1:1000, anti-TuBulin163 (#5335T) 1:5000, anti-GSDMD (#E8G3F) 1:1000, the above antibodies were purchased from Cell Signaling Technology Co., Ltd. (Danvers, MA, USA). Anti-STING (#AB288157) 1:1000, anti-MPO (#AB208670) 1:1000, and anti-Histone H3 (#AB5103) 1:1000; the above antibodies were purchased from Abcam (Cambridge, MA, USA). Anti-PAD2 (#12110-1-AP) 1:1000, anti-PAD4 (#17373-1-AP) 1:1000, the two antibodies were purchased from Proteintech (Chicago, USA). Horseradish peroxidase-conjugated goat anti-mouse antibody (H+L; A0216) and goat anti-rabbit antibody (H+L; A0208) were purchased from Beyotime Biotechnology Co., Ltd. (Shanghai, China). The BCA protein concentration determination kit was purchased from Beyotime Biotechnology Co., Ltd. (Shanghai, China). ECL ultra-sensitive luminescent liquid was purchased from Shenhua Technology Co., Ltd. (Hangzhou, China).

### Animal preparation

**Figure 1:**
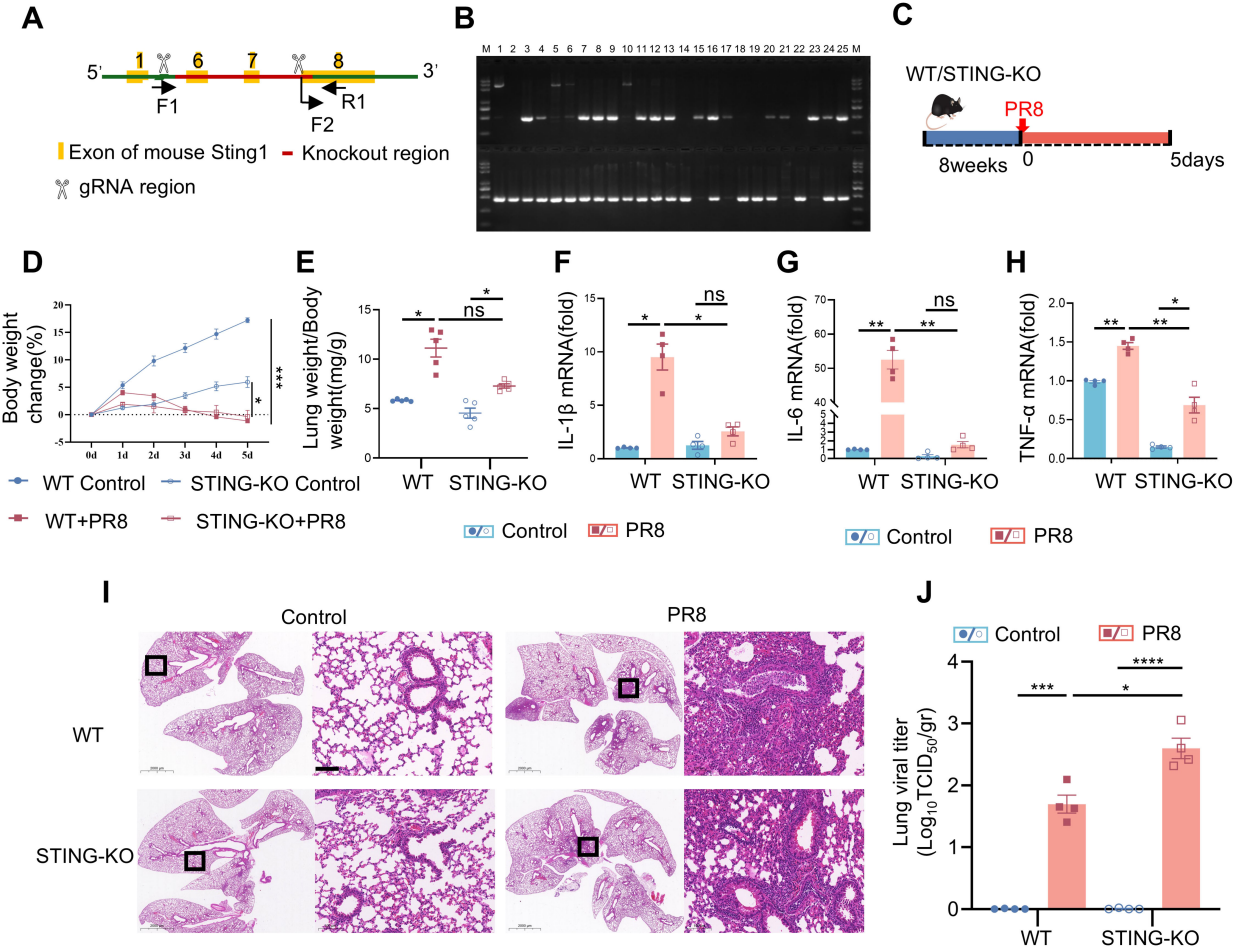
PR8-induced viral pneumonia is effectively inhibited in STING knockout mice. **(A)** Strategies for typing the STING gene. **(B)** Representative images of the identification of the STING gene. **(C)** Graphical representation of experimental models showing the effects of PR8 on WT and STING-KO mice. **(D)** Body weight change (%) (n = 10). **(E)** The lung weight index was calculated as the ratio of mouse lung weight (mg) to mouse body weight (g) (n = 5). **(F-H)** The mRNA levels of IL-1β, IL-6, and TNF-α were measured by RT-PCR. **(I)** Images of H&E staining showing notable lung pathology in WT and STING KO mice post-PR8 inhalation were acquired (scale bar: 100 μm). **(J)** Viral titration in the bronchoalveolar lavage fluid was conducted by the TCID_50_ assay (n = 4). Data are presented as the mean ± SD, *p < 0.05, **p < 0.01, ***p < 0.001, ****p < 0.0001, and ns, no significant difference between the two groups.

C57BL/6J mice (male, 6–8 weeks old, weighing 18–20 g) were purchased from the Experimental Animal Center of Hangzhou Medical College (License No: SCXK (Zhejiang) 2019-0002). STING^+/-^ mice were provided by Cyagen Bioscience (Suzhou, China; License No: SCXK (Suzhou) 2018-0003). Male and female STING^+/-^ mice were mated by a laboratory technician at the Experimental Animal Center of Hangzhou Medical College (Hangzhou, Zhejiang) to produce STING^-/-^ (STING knockout; STING-KO) mice, which were positively identified. **Figure 1A** illustrates the CRISPR/Cas9-mediated deletion of the Sting1 exon region and the design of primers F1, F2, and R1 for PCR amplification. Three primer pairs enable simultaneous discrimination of wild-type, heterozygous, and knockout alleles in a single PCR reaction. Representative genotyping results are presented in **Figure 1B**. In this study, two different animal experiments were performed. All mice were male and were housed in isolated cages at the specific pathogen-free (SPF) barrier facility of the Experimental Animal Center of Hangzhou Medical College (temperature 23 ± 2°C, relative humidity 40-60%, 12 hours light/12 hours dark cycle) with food and water provided. The experimental animals and designs complied with the requirements of the international American Association for Accreditation of Laboratory Animal Care (AAALAC) accreditation program and were approved by the Animal Ethics and Use Committee of Hangzhou Medical College (Approval 2021-029). All research on the H1N1 avian influenza virus was conducted in a biosafety level 2 laboratory.

### Induction of viral pneumonia by PR8 infection

Eighty-eight wild-type (WT) mice subjected to adaptive feeding for one week were selected for the experiment. Eight mice were randomly selected as the normal control group, and the remaining 80 mice were weakly anesthetized with 0.5% pentobarbital sodium. These eighty mice were infected with 50 μL of the PR8 influenza virus strain (80 LD_50_/mL) by nasal inhalation to establish models of viral pneumonia. Eight mice were administered prior to infection (0d) and at various intervals (0.5d, 1d, 2d, 3d, 4d, 5d, 6d, 7d, and 8d) after exposure to PR8 (n = 8). Subsequently, their lung tissues were harvested for weighing and testing.

### Evaluation of the alleviating effect of a STING inhibitor (C-176) on PR8-induced viral pneumonia

80 WT mice were randomly divided into the normal control group (Control), PR8 model group (PR8), STING inhibitor treatment group (PR8+C-176), and STING inhibitor control group (C-176) (n = 20). Mice in the normal control group and the STING inhibitor control group received intranasal administration of PBS. On day 0, mice in the PR8 and PR8+C-176 groups received 50 μL of PR8 influenza virus (40 LD_50_/mL) via intranasal administration to establish a viral pneumonia model (36). After 24 hours, the mice in the PR8+C-176 group and C-176 group were intraperitoneally injected with C-176 (750 nmol C-176 per mouse in 200 µL of corn oil; 13 mg/kg body weight), while the mice in the other two groups were given 200 µL of corn oil per mouse (37). At the dosage, C-176 effectively inhibits the STING signaling pathway without inducing significant toxicity. The treatment was repeated every 24 hours, and the mice were weighed and documented. On day 5, blood, bronchoalveolar lavage fluid (BALF), and lung tissues were collected from each group of mice. RNA was isolated from the left lung of four mice in each group, and proteins were extracted from the right lung. Five mice in each group were used for bronchiolar lavage with PBS. Lung tissue from four mice per group was utilized to determine virus titer. The remaining mice in each group were used for histopathological examination.

### Induction of viral pneumonia by PR8 in STING-KO mice

The following groups were evaluated: the WT Control group, WT+PR8 group, STING-KO Control group, and STING-KO+PR8 group (n = 20). Mice in the WT Control and STING-KO Control groups received intranasal administration of PBS. On day 0, mice in the WT+PR8 and STING-KO+PR8 groups received 50 μL of PR8 influenza virus (40 LD_50_/mL) via intranasal administration to establish a viral pneumonia model. The mice were weighed and documented daily. On the fifth day, the mice were euthanized via intraperitoneal injection of 1% sodium pentobarbital (100 mg/kg), followed by collection of lung tissues.

### Measurement of Body weight and Lung index

After weighing the mice and using day 0 as the baseline, we calculated each mouse’s percentage of weight change as follows: body weight change (%) = (daily weight – weight on day 0)/average weight on day 0 * 100%. The lungs were removed and weighed from mice euthanized via intraperitoneal injection of 1% pentobarbital sodium (100 mg/kg). The lung index was calculated as follows: lung index (mg/g) = lung weight (mg)/mouse body weight (g).

### Histopathology

Fresh lung tissue from the mice was collected and rinsed with physiological saline. The lung tissue was placed in 10% neutral buffered formalin at room temperature for fixation, and after 24 hours, the transparent tissue blocks were placed in melted paraffin and placed in a paraffin oven for infiltration. After the paraffin had completely infiltrated the tissue blocks, they were embedded and cooled to solidify the paraffin. The paraffin-embedded blocks were sliced at a thickness of 5-8 μm, and the sections were deparaffinized and stained with hematoxylin and eosin (H&E). Observe and image the stained samples using an optical microscope.

### Collection and analysis of bronchoalveolar lavage fluid

Five mice were taken from each group. After euthanasia, the chest was opened to expose the trachea. A catheter was inserted into the trachea, and 1 mL of physiological saline was obtained using a syringe. The catheter was connected, and the saline was pushed into the lungs through the trachea. After three lavages, the lavage fluid was extracted and added to a 1.5 mL centrifuge tube. The tube was centrifuged at 4°C and 3000 r/min for 5 minutes in a chilled centrifuge. Cells were collected and washed with pre-chilled PBS. Cells were resuspended in 100 µL PBS, 50 µL of the resulting suspension was pipetted onto a microscope slide and spread evenly, allowed to air-dry, and then fixed with formaldehyde. The slide was stained with Wright-Giemsa solution for 1 minute, then an equal volume of pH 6.8 buffer for 5 minutes. The slide was rinsed gently with distilled water and allowed the microscope slide to air-dry. Total cells, neutrophils, and macrophages were observed and counted under a light microscope. Cell counting was independently performed by researchers in a single-blind manner.

### Virus isolation and titration

Mouse lung tissues were immersed in MEM (Gibco, Thermo Fisher, Canada), homogenized using a TissueLyser II (Qiagen, Hilden, Germany) at 25 Hz for 5 minutes, followed by centrifugation. The homogenized lung samples and BALF supernatants were then sucked out and stored. Virus titers were calculated using the Spearman-Karber algorithm and measured by the 50% tissue-culture infective dose (TCID50) assay (38, 39).

### Quantitative fluorescence reverse transcription polymerase chain reaction

One hundred milligrams of lung tissue were collected, and total RNA was extracted using a TRIzol reagent. The purity and concentration of the RNA were determined by measuring the absorbance at 260 nm and 280 nm. The extracted RNA was reverse transcribed into cDNA using PrimeScript RT Master Mix (RR036A, Takara, Shiga, Japan), and the cDNA was subjected to qPCR using MonAmpTM SYBR Green qPCR Mix (Mona) in a real-time PCR system (Applied Biosystems, Foster City, California, USA). GAPDH was used as the reference gene. The relative expression levels of target genes, including IL-6, IL-1β, TNF-α, IFN-β, ISG15, and CXCL10, in the lung tissue were calculated using the 2^-ΔΔCt^ method. All primers were designed and were listed in the **Supplementary Table S1**, and the sequences are available in GenBank (https://www.ncbi.nlm.nih.gov/genbank/).

### Western blot analysis

Mouse lung tissue (100 mg) was collected and homogenized using a homogenizer in RIPA lysis buffer (Beyotime Biotech, Shanghai, China). The homogenate was then centrifuged at 12,000 × g and 4°C for 15 minutes, and the supernatant was collected for total protein quantification using a BCA assay kit (Beyotime Biotech, Shanghai, China). The remaining supernatant was subjected to SDS-PAGE on a 10% polyacrylamide gel to separate the proteins, which were then transferred to a PVDF membrane. The membrane was blocked with 5% skim milk and incubated overnight with the corresponding primary antibody at 4°C. The following day, after washing with TBST, the membrane was incubated with an HRP-conjugated secondary antibody for 1 hour. Immunoreactive protein bands were detected using an ECL detection kit, and grayscale values were analyzed using ImageJ software (Version 1.53u, Wayne Rasband, USA). Anti-GAPDH and anti-Tubulin were used as reference standards for protein quantification.

### Enzyme-linked immunosorbent assay

The content of mouse myeloperoxidase-DNA (MPO-DNA) complexes associated with NETs in BALF was measured. This method utilized several reagents from the MPO-DNA ELISA kit (Meimian, Jiangsu, China). First, accurately quantified samples were added to a 96-well ELISA microplate coated with the enzyme label (including blank wells, standard wells, and test sample wells) and gently mixed, and the microplate was then incubated at 37°C for 30 minutes. After five washes, the enzyme label reagent was added for incubation at 37°C for 30 minutes. After five washes, the chromogenic solution was added for incubation at 37°C for 10 minutes, and the stop solution was then added. The absorbance (OD value) at 450 nm of each well was measured sequentially. The sample concentration was calculated based on the standard curve.

### Immunofluorescence staining of lung tissue

Mouse lung tissue was removed, fixed with 4% formaldehyde solution for 24 hours, dehydrated, paraffin-embedded and sectioned. The sections were deparaffinized, repaired with citrate, blocked with 5% goat serum solution, washed, and incubated with the corresponding primary antibody overnight. The samples were washed with 0.25% PBST 3 times for 5 minutes each. A fluorescent secondary antibody staining solution was prepared with a diluted fluorescent secondary antibody against the corresponding species, and the sections were incubated for 1 hour at room temperature in the dark. The sections were then washed with PBST 3 times for 5 minutes each. The sections were stained with DAPI, mounted, and examined under a fluorescence microscope (Olympus, Japan).

### Immunohistochemical staining of STING in lung tissue

Sections were subjected to immunohistochemical staining with the SP method, routine dewaxing, antigen retrieval, primary antibody (anti-STING) addition, and incubation at 4°C overnight. The next day, the secondary antibody was added dropwise, followed by colorization with DAB and contrast staining with hematoxylin.

### Transcriptomics analysis

Total RNA was extracted using a TRIzol kit and tested for purity using a NanoPhotometer^®^ spectrophotometer, while the integrity and concentration of the RNA samples were tested using an Agilent 2100 RNA Nano6000 Assay Kit for quality control. The samples were then sequenced and filtered based on the Illumina platform using the PE150. Analysis of differentially expressed genes (DEGs) between the control and virus-exposed groups was performed by DESeq2, and the identification of DEGs was based mainly on the fold change (FC) value and q-value (corrected P value), with the criteria of |log2FC|≥1 and q<0.05.

### Immunoprecipitation assay

Coimmunoprecipitation (CoIP) was used to investigate the interactions between the purified and enriched target proteins. Total protein was extracted using mild lysis buffer (20 mM Tris/HCl, pH 7.6; 150 mM NaCl; 20 mM KCl; 1.5 mM MgCl2; 0.5% NP-40; and 0.5 M PMSF). The antibody was incubated with Protein A/G beads for 2–3 hours, and the supernatant was then added, resulting in the formation of bead–protein A/G–antibody–target protein complexes. The mixture was then incubated overnight at 4°C. The next day, the samples were washed with TBST buffer containing 0.1% Tween 20 and subjected to immunoblot analysis after heating in a metal bath (TU-10, BIOER, Hangzhou, China) at 95°C for 5 minutes. Rabbit polyclonal IgG (10500 C, Invitrogen, CA, USA) was used to identify false-positive binding.

### Isolation and processing of mouse neutrophils

Mice were euthanized via intraperitoneal injection of 1% pentobarbital sodium (100 mg/kg), and the femurs and tibias were aseptically harvested and placed in sterile Petri dishes. The callus was subsequently dissected, and the marrow cavity was flushed with complete culture medium (RPMI 1640 medium supplemented with 10% fetal bovine serum, 1% penicillin-streptomycin, and 2 mM EDTA) and filtered through a sterile 200-mesh nylon reticulocyte filter. Next, the bone marrow cell suspension was combined with neutrophil separation buffer (CB7701, G-CLONE, Beijing, China) in a 15 mL centrifuge tube and subjected to centrifugation at 1000 × g for 30 minutes at room temperature. After centrifugation, the opaque white cell layer at the center of the centrifuge tube was isolated, and the red blood cells were lysed using red cell lysis buffer, with the remaining fraction designated the neutrophil fraction.

This study involved the isolation of WT and STING-KO neutrophils for distinct treatments. In the initial trial, WT neutrophils were divided into three groups (control, PR8, and PR8+C-176) and distributed in six-well plates. Neutrophils in the PR8 and PR8+C-176 groups were stimulated with PR8 to establish an *in vitro* model of NETs, and a STING inhibitor (C-176) was added to the PR8+C-176 group. Following a 6-hour treatment period, the neutrophils were fixed, permeabilized, and blocked. After overnight incubation with primary antibodies at 4°C, the cells were washed three times with PBST and incubated with the corresponding species-specific fluorescent secondary antibody for 1 hour at room temperature in the dark. Following three subsequent washes with PBST, the cells were incubated with primary antibodies and fluorescent secondary antibodies conjugated to different colors of fluorophores and targeting a species different from the previously targeted species. Nuclei were stained with DAPI, the sections were mounted, and images were acquired with a fluorescence microscope (Zeiss Axiovert 200, Germany). In the subsequent experiment, WT and STING-KO neutrophils were divided into two groups (control and PR8) and subjected to PR8 induction and immunofluorescence imaging employing the same technique used in the previous experiment.

### Statistical analysis

Statistical analysis was performed using GraphPad software (version 9.5) with a significance level of p<0.05. The sample size (N) for each experiment was determined by the number of replicates with three or more experiments. The data are presented as the mean ± standard deviation values. One-way analysis of variance (ANOVA) and intergroup comparisons were used for comparisons of continuous variables between different pairs of groups.

## Results

### Induction of viral pneumonia by PR8 infection

**Figure 2:**
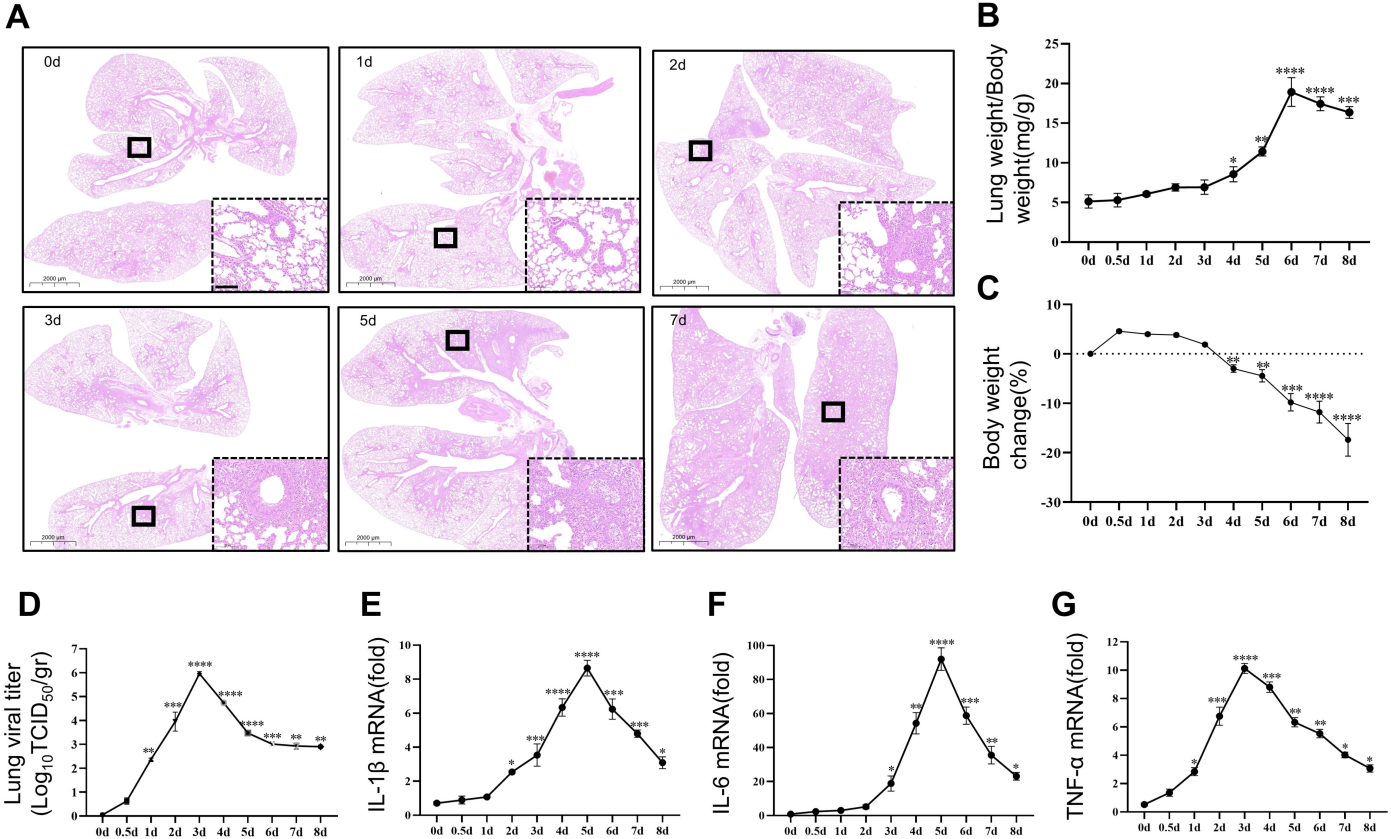
Induction of viral pneumonia by PR8 infection. Comparison of the severity of viral pneumonia according to the duration of viral action in mice. **(A)** Representative images of pathological changes in lung tissue from C57BL/6J mice stained with hematoxylin and eosin (scale bar: 100 μm). **(B)** The lung indices in mice exposed to the PR8 virus were calculated by the following equation: lung index = mouse lung weight (mg)/mouse body weight (g) (n = 8). **(C)** Body weight change (%) (n = 8). **(D)** Viral titration of homogenized lung samples was conducted by the TCID_50_ assay. The samples were analyzed in triplicate, and each dot corresponds to the viral titers of an individual mouse lung (n = 3). **(E-G)** The expression of the inflammatory cytokines IL-1β, IL-6, and TNF-α in lung tissue homogenates was measured by RT–qPCR (n = 3). Data are presented as the mean ± SD, *p < 0.05, **p < 0.01, ***p < 0.001, and ****p < 0.0001 vs. 0day.

To investigate the temporal effect of viral pneumonia, we conducted experiments in mice to investigate the relationship between the time after inhalation of the PR8 virus and the severity of viral inflammation. Beginning on the second day of infection, the alveolar structure in the lung tissue was significantly disrupted, and inflammatory cell infiltration became more pronounced, peaking on the fifth day (**Figure 2A**). The body weight of the animals decreased significantly after 4 days of infection (**Figure 2C**), while the relative weight of the lungs increased significantly after 4 days of infection, suggesting the development of pulmonary edema (**Figure 2B**). Viral titration in lung tissue was found to be significant on the first day and peaked on the third day (**Figure 2D**). Notably, the release of inflammatory factors (IL-1β, IL-6, and TNF-α) in the lungs of the mice increased significantly on day 2 and peaked on day 5 (**Figures 2E–G**). However, the levels of inflammatory factors gradually decreased in the later stages of viral infection.

### Transcriptomic analysis revealed that STING is a potential therapeutic target for viral pneumonia

**Figure 3:**
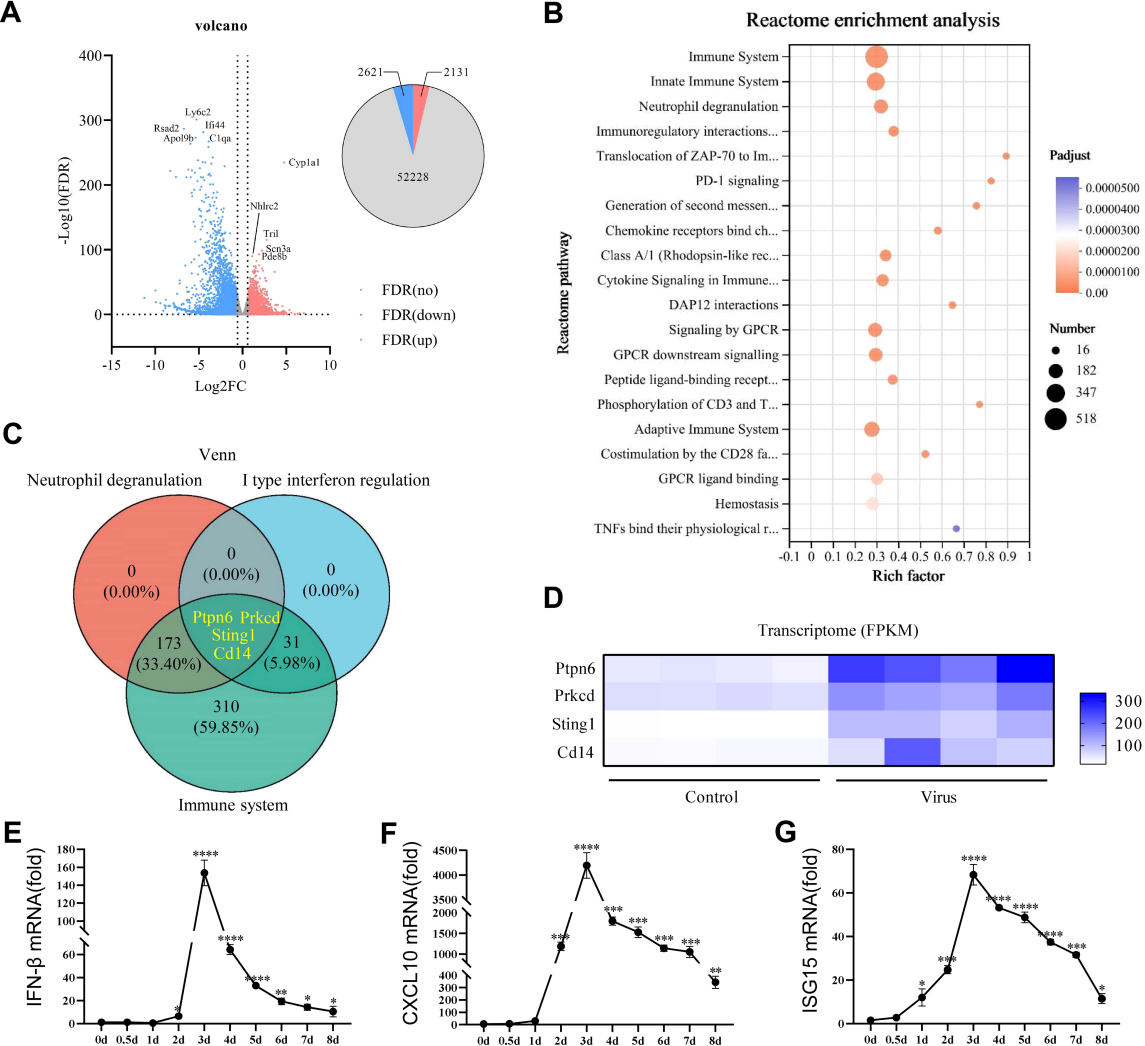
STING was identified as a potential therapeutic target for viral pneumonia in histological analysis. **(A)** Volcano plot showing the expression profiles of the top ten upregulated and downregulated DEGs in the lung tissues of mice on the fifth day of exposure to PR8. The upregulated genes were identified using false discovery rate (FDR) correction with log2FC>1 as the criterion is highlighted in red. **(B)** Bubble plot illustrating the results of Reactome enrichment analysis of the significant DEGs in the lungs of mice on the fifth day of exposure to PR8, with log2 fold change >2 and adjusted p<0.01 as the criteria. **(C)** Venn diagram displaying the numbers and proportions of overlapping type I IFN, neutrophil degranulation, and immune system-related genes among the DEGs related to viral pneumonia. **(D)** Heatmap showing the results of correlation analysis of key genes identified in the Venn diagram between the Control group and model group. DEGs, differentially expressed genes. **(E-G)** RT–qPCR analysis of the mRNA expression of IFN-β, CXCL10, and ISG15 in mouse lung tissues (n = 3). Data are presented as the mean ± SD, *p < 0.05, **p < 0.01, ***p < 0.001, and ****p < 0.0001 vs. 0day.

Severe viral lung inflammation occurred 5 days after PR8 infection (**Figure 2E**). Therefore, transcriptomic analyses were conducted on mouse lung tissue at this specific time point to gain deeper insights into the alterations in inflammatory signaling pathways triggered by viral infection in mouse lungs. The volcano plot revealed 4752 DEGs after PR8 intervention: 2131 upregulated genes and 2621 downregulated genes (**Figure 3A**). The top 10 upregulated and downregulated genes are labeled in **Figure 3A**. To explore the biological functions and pathways of the DEGs, Reactome enrichment analysis was conducted. Reactome enrichment analysis of the DEGs revealed that the PR8-regulated genes were involved mainly in the immune system, type I IFN signaling, and neutrophil degranulation signaling (**Figure 3B**). Subsequently, we conducted a Venn diagram analysis of the immune-related, type I IFN-related, and neutrophil degranulation-related genes among the DEGs (**Figure 3C**). This analysis identified Sting, Ptpn6, Prkcd, and Cd14 as key genes involved in viral pneumonia (**Figure 3D**). As STING is an important gene involved in the response of the immune system to viral invasion (40), we guessed that reducing the progression of viral pneumonia by interfering with the STING signaling pathway. We measured the mRNA levels of STING-induced type I IFN-related factors (IFN-β, CXCL-10, ISG15). Expectably, the mRNA expression levels of type I IFN-related factors were significantly increased on day 1 and peaked on day 3 after PR8 infection (**Figures 3E–G**). This temporal pattern differed from that of the expression levels of inflammatory factors. Therefore, targeting STING signaling may be an effective treatment for viral pneumonia induced by PR8, and STING inhibition is planned to be initiated 24 hours after viral infection to reduce viral inflammation.

### Pharmacological blockade of STING has a protective effect against PR8-induced viral pneumonia

**Figure 4:**
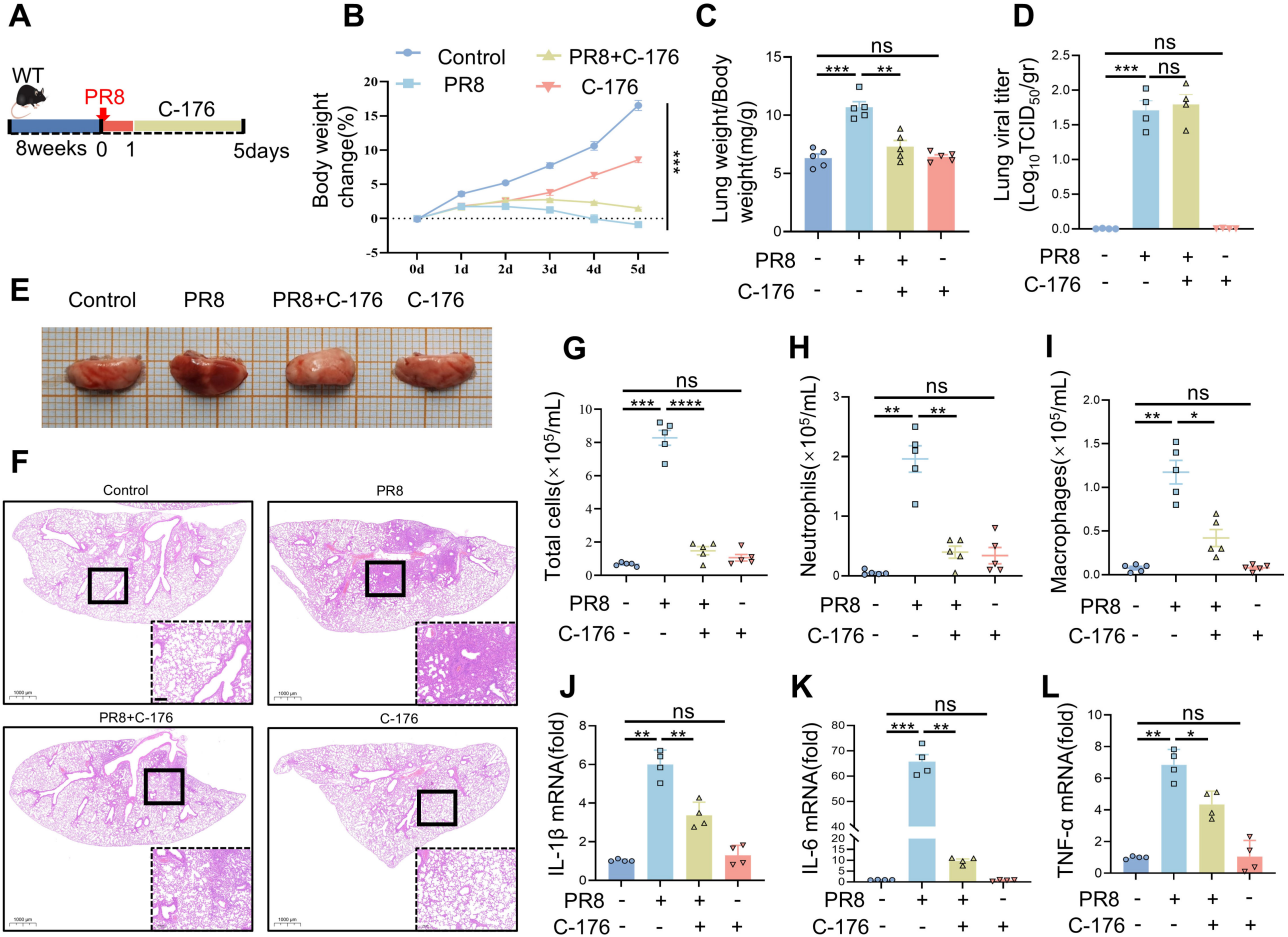
The pharmacological blockade of STING has a protective effect against PR8-induced viral pneumonia. **(A)** The experimental protocol involved the induction of viral pneumonia *in vivo* using the PR8 strain (15 mice per group). **(B)** Body weight change (%) (n = 15). **(C)** The lung weight index was calculated as the ratio of lung weight (mg) to body weight (g). **(D)** Viral titration in the bronchoalveolar lavage fluid was conducted by the TCID_50_ assay (n = 4). **(E)** Representative gross images of lung tissue from each group were examined. **(F)** H&E-stained lung sections from the Control, PR8, PR8+C-176, and C-176 groups were analyzed (scale bar: 200 μm). The Control and C-176 groups exhibited a normal lung structure, characterized by an intact ciliated airway epithelium and alveoli. In contrast, influenza infection led to severe bronchiolitis, inflammation, and alveolar damage, which were alleviated by treatment with C-176. **(G-I)** The numbers of total cells, neutrophils, and macrophages in the bronchoalveolar lavage fluid were determined (n = 5). **(J-L)** The mRNA levels of IL-1β, IL-6, and TNF-α in the lung tissues of mice in the four groups were measured using RT-PCR (n = 4). Data are presented as the mean ± SD, *p < 0.05, **p < 0.01, ***p < 0.001, ****p < 0.0001, and ns, no significant difference between the two groups.

To determine whether STING serves as a target for treating viral pneumonia, STING inhibition was performed in WT mice. Mice were infected with PR8 and injected intraperitoneally with STING inhibitors, as shown in the experimental flowchart in **Figure 4A**. Throughout the experiment, all four mouse groups exhibited changes in body weight. Significant weight differences were observed between the Control and PR8 groups (**Figure 4B**). Treatment with C-176 demonstrated notable efficacy in mitigating PR8-induced pulmonary edema, as illustrated in **Figure 4C**. The viral titers in the lungs of mice infected with PR8 was significantly higher than in the Control group. However, no significant difference was observed between the PR8 group and the PR8+C-176 group (**Figure 4D**). The lung tissues in the PR8 group appeared congested and reddish, with a small increase in volume. These effects were significantly ameliorated after C-176 treatment (**Figure 4E**). Furthermore, the injection of compound C-176 reduced the infiltration of inflammatory factors induced by PR8 (**Figure 4F**) and decreased the levels of proinflammatory factors (IL-1β, IL-6, and TNF-α) in the lung tissue (**Figures 4J–L**). Total cells, neutrophils and macrophages counts were significantly higher in the BALF of the model group mice than in the control group. However, these counts were significantly lower in the BALF after C-176 treatment than in the model group (**Figures 4G–I**).

Activation of STING proteins activates the NF-κB and IRF3 downstream signaling pathways, which play a crucial role in antiviral immunity. We analyzed the expression of mRNAs and proteins related to the STING and NF-κB pathways after treatment with C-176. The results showed significant increases in the mRNA expression levels of IFN-related factors (IFN-β, CXCL10, and ISG15) in the lungs of mice infected with PR8 (**Supplementary Figures S1A–C**). However, inhibiting STING (the PR8+C-176 group) significantly reduced the mRNA expression levels of IFN-related factors compared to those in the PR8 group (**Supplementary Figures S1A-C**), consistent with the changes in the mRNA expression levels of proinflammatory factors (**Figures 4J–L**). Additionally, the phosphorylation levels of TBK1, NF-κB, and IRF3 were significantly lower in the PR8+C-176 group than in the PR8 group (**Supplementary Figure S1D**), with a statistically significant difference (**Supplementary Figure S1E**) (*p* < 0.05). The result of immunohistochemistry showed that the expression of STING protein was significantly increased in the lung tissues of PR8-infected mice compared to the control group (**Supplementary Figure S1F**). In contrast, the PR8+C-176 group exhibited low STING expression and decreased inflammation (**Supplementary Figure S1F**), indicating that the inhibition of STING may be useful for the treatment of viral pneumonia induced by PR8. In other words, the inhibition of STING can act as an anti-inflammatory target, effectively preventing viral pneumonia-associated injury.

### PR8-induced viral pneumonia is effectively inhibited by STING deficiency

To further elucidate the role of STING in regulating viral pulmonary inflammation, we infected STING-KO mice with PR8 and evaluated various indices of viral pneumonia. The typing strategy for confirming STING gene deletion is shown in **Figure 1A**. STING^-/-^ mice were identified after crossbreeding using methods and reagents provided by Cytec Biologics (Cyagen). Representative images of the identified mice showed that numbers 1, 2, 5, 6, 10, 14, 18, 19, and 22 were WT mice; numbers 15, 17, 21, and 23 were STING^-/-^ mice; and the remaining mice were STING^+/-^ mice (**Figure 1B**). Then, STING-KO mice and WT mice were infected with the PR8 influenza virus, and weight and survival were assessed for 5 days (**Figure 1C**). Following infection with PR8, the body weight loss and pulmonary edema did not differ significantly between WT mice and STING-KO mice (**Figures 1D, E**). However, the PR8 model group of WT mice exhibited more severe alveolar structural damage and inflammatory cell infiltration than did the PR8 group of STING-KO mice (**Figure 1I**). Notably, viral inflammation was less likely to be caused by PR8 infection in STING-KO mice than in WT mice. RT-PCR analysis demonstrated that the absence of STING led to decreases in the mRNA levels of inflammatory mediators (IL-1β, IL-6, and TNF-α) induced by PR8 (**Figures 1F–H**), as well as IFN-related factors (IFN-β, CXCL10, and ISG15) (**Supplementary Figures S2C-E**). Additionally, the absence of STING attenuated the phosphorylation of TBK1, NF-κB, and IRF3 induced by PR8 (**Supplementary Figures S2F-G**). However, following PR8 infection, STING-KO mice exhibited significantly higher viral titers in the lungs compared to WT mice (**Figure 1J**). Overall, the above findings revealed that STING signaling plays a crucial role in the development of viral pneumonia induced by PR8. Targeting the STING pathway represents a potential therapeutic strategy for viral pneumonia.

### The formation of NETs in PR8-induced viral pneumonia is affected by STING inhibition

**Figure 5:**
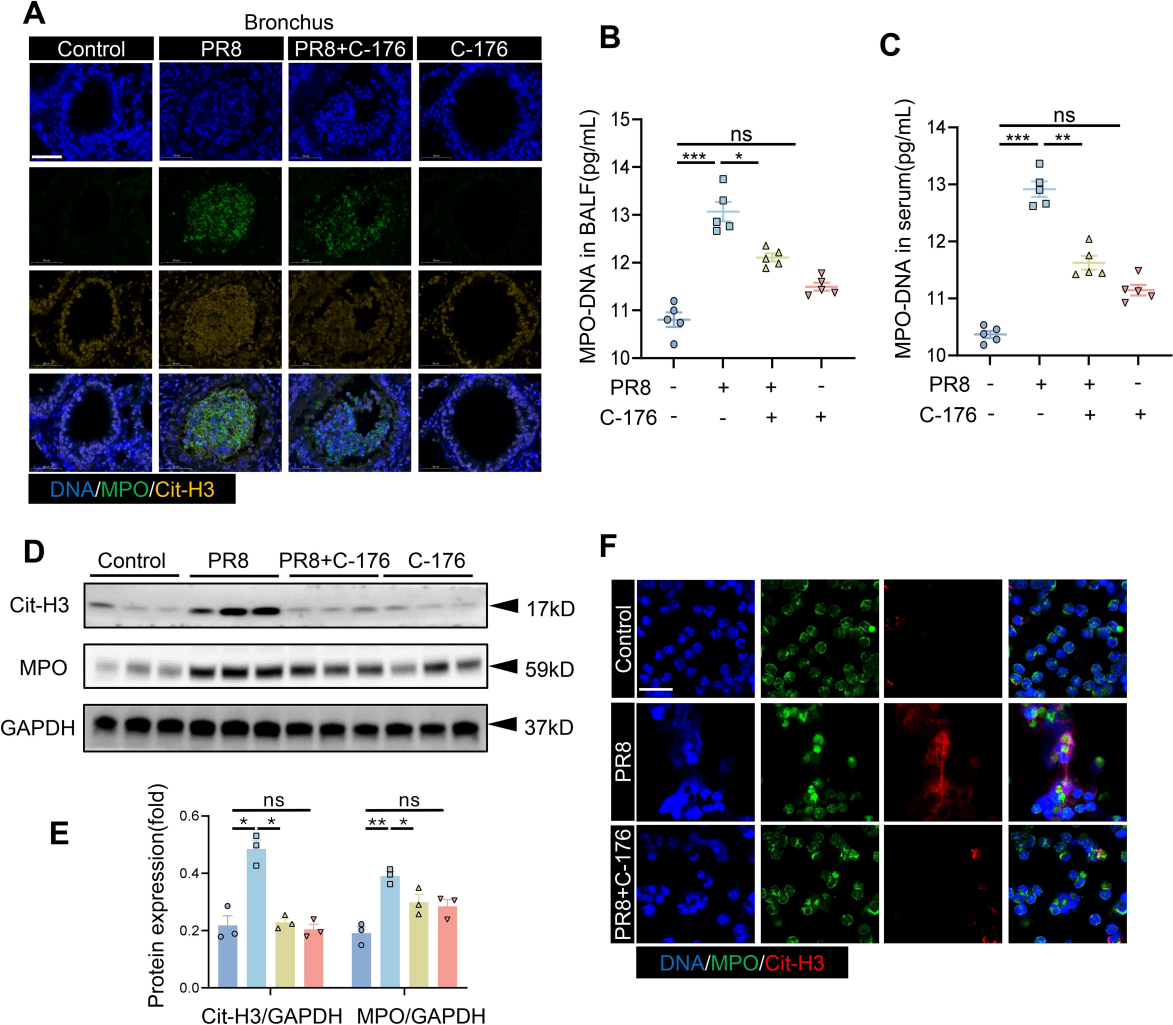
The production of NETs in PR8-induced viral pneumonia is affected by STING inhibition. **(A)** Lung tissue sections from mice in the four groups (Control, PR8, PR8+C-176, and C-176) were examined by immunofluorescence staining, as shown in the images (scale bar: 50 μm). MPO is labeled in green, and Cit-H3 is labeled in yellow. The concentrations of MPO-DNA complexes (expressed in pg/mL) in the BALF **(B)** and serum **(C)** of mice in all the experimental groups were quantified utilizing ELISA (n = 5). **(D)** Western blot analysis was conducted to evaluate the protein expression of MPO and Cit-H3 relative to that of GAPDH, which was used as a loading control (n = 3). **(E)** Band densities were quantified for graphical representation using ImageJ software and normalized to those of GAPDH. Data are presented as the mean ± SD, *p < 0.05, **p < 0.01, ***p < 0.001, ****p < 0.0001, and ns, no significant difference between the two groups. **(F)** Neutrophils were infected with 1ul of the PR8 virus strain (40 LD_50_/mL) and treated with C-176 (200 nM) *in vitro*. Subsequent visualization was performed using laser confocal microscopy, revealing the presence of MPO (green) and Cit-H3 (red) in the acquired images (scale bar: 10 µm).

Our analysis of the transcriptomic data focused on the association of neutrophil degranulation signaling with viral pneumonia (**Figure 3B**). Neutrophils are recruited and then degranulate to release DNA and antimicrobial proteins in order to form NETs, which capture and degrade pathogenic microorganisms. MPO and citrullinated histones (Cit-H3) are known biomarkers of NETs. Considering the association between viral pneumonia development and NETs, NETs-related studies were conducted on alveoli and bronchi in a mouse model of viral pneumonia. Interestingly, significant citrullination was observed in both the bronchi and alveoli of the viral pneumonia model mice, suggesting the involvement of NETs in viral pneumonia. However, NETs formation was reduced when STING was inhibited. (**Figure 5A**, **Supplementary Figure S2A**). The inhibitors of STING (C-176 group) had no significant impact on NETs formation in the lungs of mice (**Figure 5A**). The potential association between STING and NETs formation in viral pneumonia attracted our attention. The levels of MPO-DNA complexes were increased in the serum and BALF following viral infection but decreased after the administration of C-176 (**Figures 5B, C**). Western blot analysis of NET-related proteins in lung tissue revealed significant increases in MPO and Cit-H3 protein levels after viral infection compared to those in the control group. However, the protein expression levels of MPO and Cit-H3 were significantly lower in the treated group than in the model group (**Figures 5D, E**). Furthermore, we isolated neutrophils from WT mice for *in vitro* infection with PR8 and added C-176 to modulate the infection process. Our observations revealed a notable increase in the formation of NETs by neutrophils following PR8 exposure, which subsequently decreased upon STING inhibition (**Figure 5F**). These results indicate that STING is an important factor in the formation of NETs during PR8-induced viral pneumonia.

### STING regulates the formation of NETs in viral pneumonia

**Figure 6:**
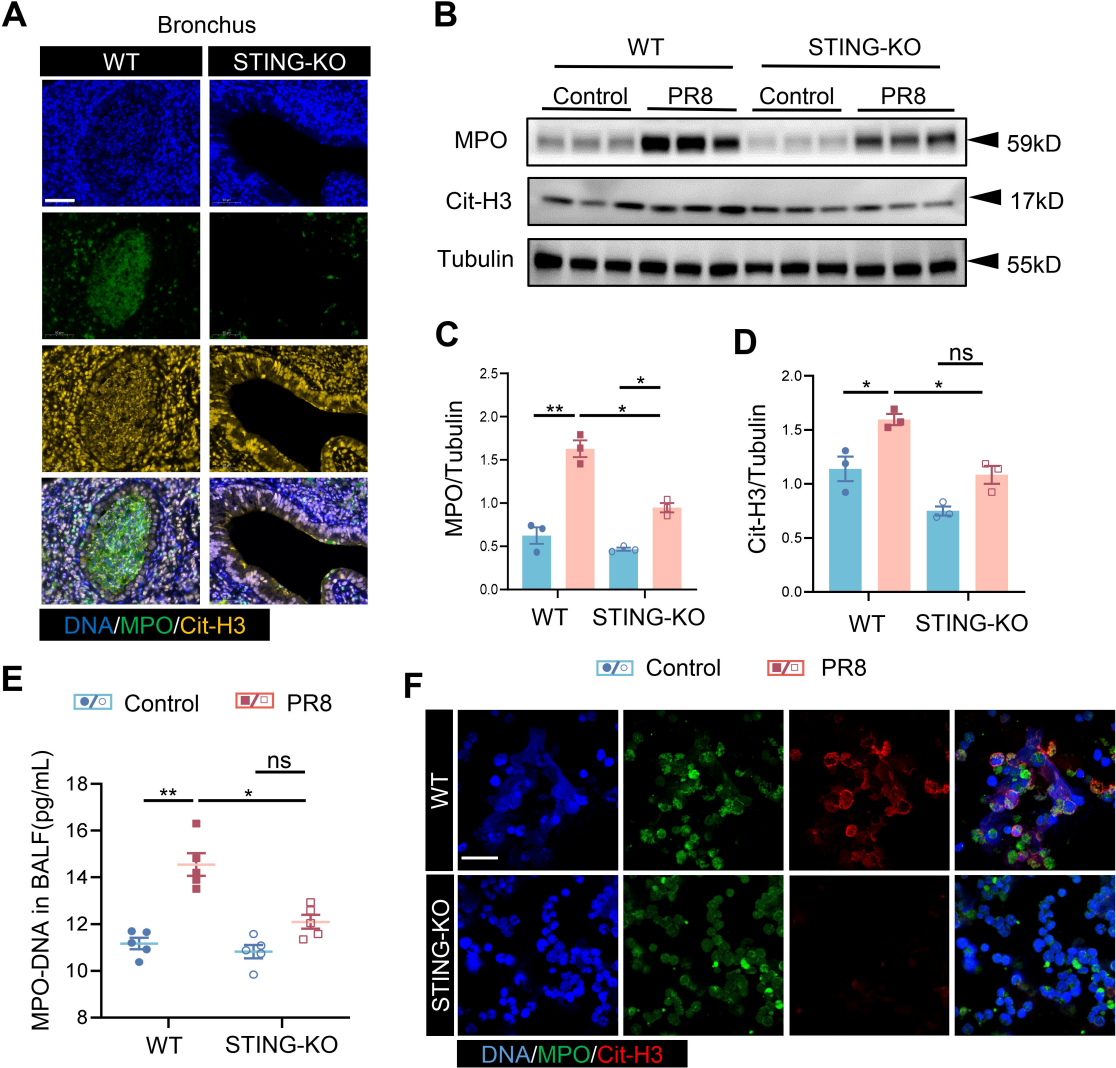
STING regulates the generation of NETs in viral pneumonia. **(A)** PR8-induced formation of NETs in the bronchi of WT and STING-KO mice was identified through immunofluorescence analysis (scale bar: 50 μm). MPO is labeled in green, and Cit-H3 is labeled in yellow. **(B)** MPO and Cit-H3 protein expression levels were measured via Western blot analysis, utilizing Tubulin as an internal control (n = 3). **(C, D)** Quantification of B-figure was performed using ImageJ software with a sample size of three. **(E)** The concentration of MPO-DNA complexes in BALF, measured in picograms per milliliter (pg/mL), was assessed in each group of mice (n = 5) using an enzyme-linked immunosorbent assay (ELISA). Data are presented as the mean ± SD, *p < 0.05, **p < 0.01, ***p < 0.001, ****p < 0.0001, and ns, no significant difference between the two groups. **(F)** Following infection with PR8, neutrophils isolated from both WT and STING-KO mice were examined for the formation of NETs through immunofluorescence staining (scale bar: 10 μm). MPO is labeled in green, and Cit-H3 is labeled in red.

After establishing that the formation of NETs is increased in PR8-induced viral pneumonia and decreased with STING inhibition, an investigation was conducted to determine whether the mechanism of NETs formation in viral pneumonia is regulated by STING. The co-expression of MPO and Cit-H3 (markers for NETs) was lower in the bronchi and alveoli of STING-KO mice infected with PR8 than in those of the counterpart WT mice (**Figure 6A**, **Supplementary Figure S2B**), indicating that the activation of STING promotes the formation of NETs in viral pneumonia. Similarly, the total protein levels of MPO and Cit-H3 in the lung tissues of mice in the STING-KO model group were significantly lower than those in the lung tissues of mice in the WT model group (**Figures 6B–D**). The level of MPO-DNA complexes in the BALF of PR8-infected STING-KO mice was also noticeably lower than that in the BALF of PR8-infected WT mice (**Figure 6E**). Notably, neutrophils were isolated from WT and STING-KO mice, and indicators of NETs formation were detected following PR8 infection of the neutrophils. NETs released by neutrophils from STING-KO mice were significantly lower than those of NETs released by neutrophils from WT mice after viral infection of both types of neutrophils (**Figure 6F**). These findings confirm that the absence of the STING gene inhibits the formation of NETs, thereby protecting against viral pneumonia. Thus, our findings indicate that excessive activation of STING signaling promotes the formation of NETs, consequently aggravating viral pneumonia.

### STING regulates NETs formation by activating GSDMD in influenza viral pneumonia

**Figure 7:**
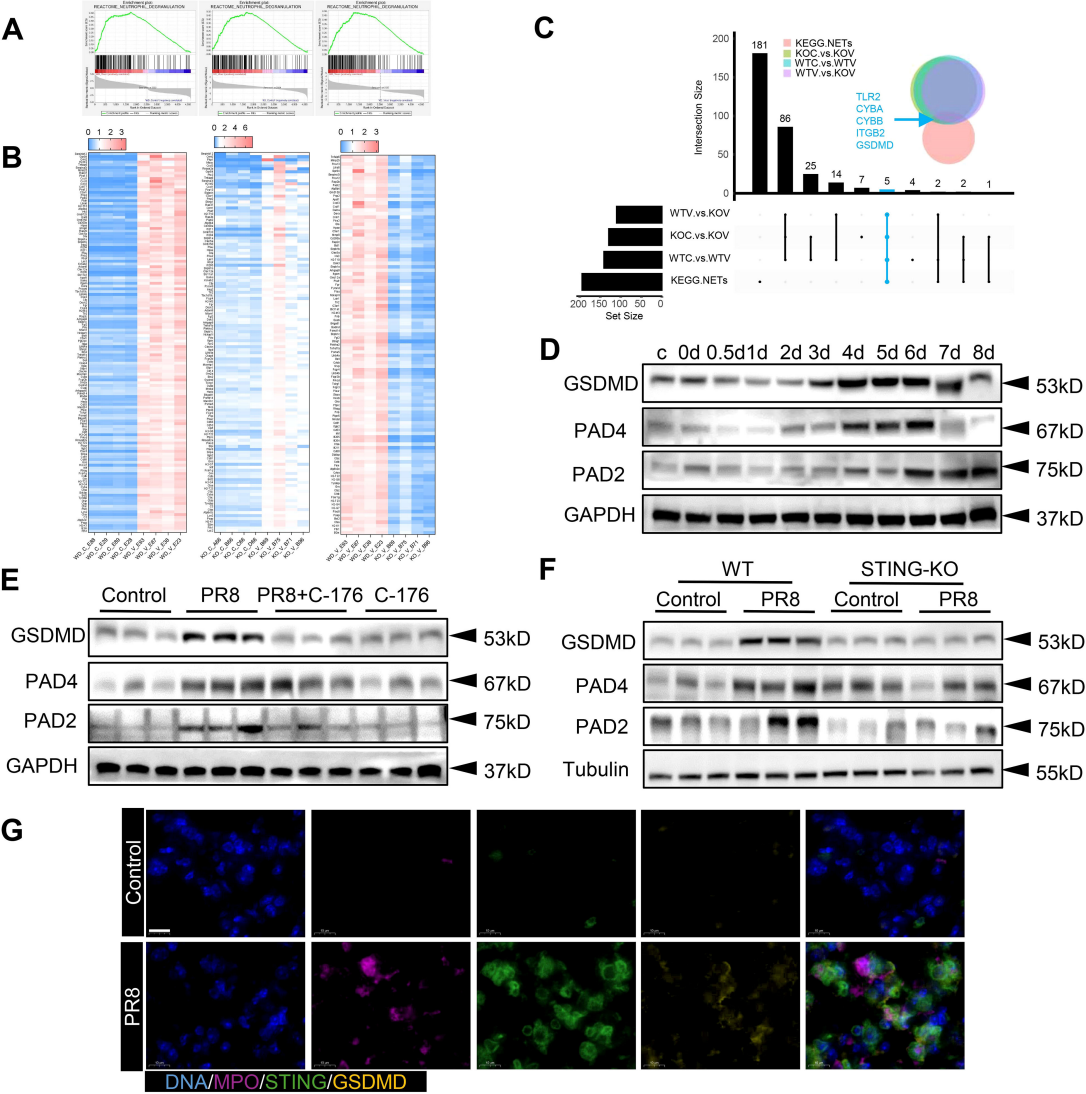
The regulation of NETs formation by STING is related to GSDMD in viral pneumonia. **(A)** Lung tissues from mice in the four groups (WT Control, WT+PR8, STING-KO Control, and STING-KO+PR8) were subjected to transcriptomic analysis. As determined by GSEA of the dataset, the vertical axis values on the “enrichment scores line graph” for the three groups were all above 0, indicating upregulation of the gene set (pathway) in the left group. **(B)** The heatmap shows the genes that directly corresponded to the gene set in panel **(A)**. **(C)**. A pairwise interaction analysis was conducted between NETs-related targets in the KEGG database and the genes identified by GSEA of the animal transcriptome. **(D-F)** In the *in vivo* experiments, the protein levels of GSDMD, PAD4, and PAD2 were measured via immunoblotting (n = 3). **(G)** An immunofluorescence assay was used to visualize the protein expression of STING and GSDMD on neutrophils in the lung tissue from the WT mice in the Control and PR8 groups (scale bar: 10 μm). MPO is labeled in purple, STING is labeled in green, and GSDMD is labeled in yellow.

The above results indicate that the formation of NETs in PR8-induced viral pneumonia is affected by STING. Therefore, further exploration of the molecular mechanisms through which the formation of NETs in viral pneumonia is regulated by STING signaling was necessary. Transcriptomic analysis was conducted on four groups of mouse lung tissues: WT Control, WT+PR8, STING-KO Control, and STING-KO+PR8. The results of gene set enrichment analysis (GSEA) and heatmap analysis showed that knockout of STING downregulated the expression of NETs-related genes (**Figures 7A, B**). Moreover, a two-by-two interaction analysis was performed using the KEGG database of NETs-related targets and the genes identified by GSEA of our animal transcriptomic data. The analysis revealed five target genes, namely TLR2, CYBA, CYBB, ITGB2, and GSDMD (**Figure 7C**). Notably, the formation of NETs is dependent on defects in or disruption of the neutrophil plasma membrane. Unsurprisingly, GSDMD is a pore-forming effector protein that is cleaved to mediate cell membrane permeabilization, and the protein encoded by the GSDMD target gene plays a role in targeting the plasma membrane to induce pore formation (31). Specific functions of GSDMD in neutrophils, such as mediating the formation of NETs in transfusion-associated acute lung injury and novel coronavirus infections, have been identified (41). Therefore, we hypothesized that GSDMD activation is the biological condition under which NETs formation is regulated by STING. Additionally, PAD2 and PAD4 not only catalyze histone citrullination but also act as chromatin densification agents, which are important components of NETs (42). We analyzed the expression of these NETs-related proteins (PAD4 and PAD2) and the GSDMD protein in lung tissues from the mice used in the previous *in vivo* experiments. The GSDMD, PAD4 and PAD2 expression levels exhibited an increasing trend with increasing virus infection time (**Figure 7D**). However, their protein expression levels decreased significantly after treatment with C-176 (**Figure 7E**). Furthermore, the expression of GSDMD and NETs-related proteins in the lungs of the mice in the STING-KO model group was significantly lower than that in the lungs of the mice in the WT model group (**Figure 7F**). Considering the above results collectively, we determined that the expression trends of STING, GSDMD and NETs-related proteins were positively correlated *in vivo*. Moreover, the results of immunofluorescence experiments showed that the expression of both STING and GSDMD in NETs formed in the lungs of WT mice infected with PR8 was upregulated (**Figure 7G**). These results indicate that both STING and GSDMD are involved in the formation of NETs in PR8-induced viral pneumonia.

**Figure 8:**
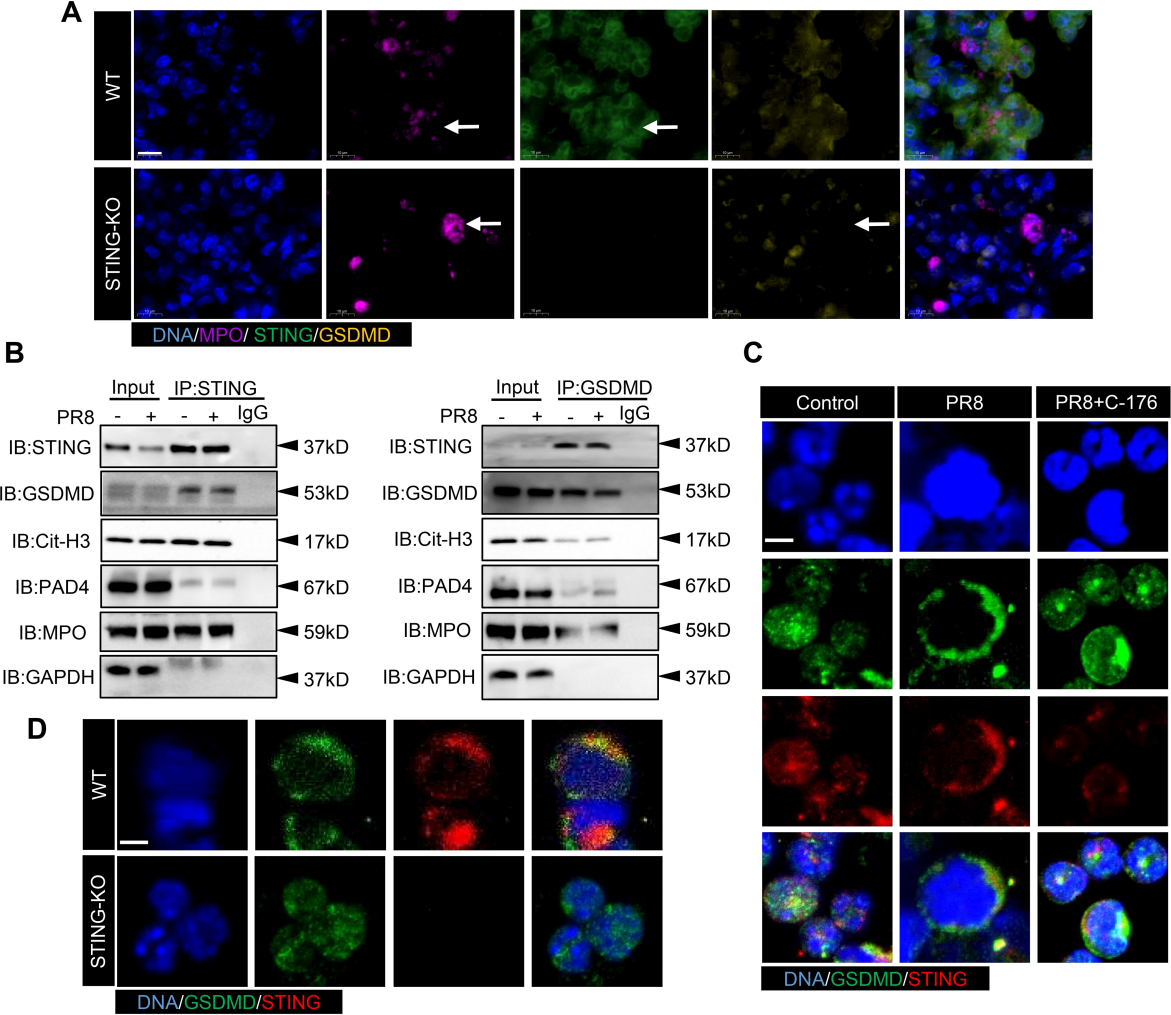
The activation of GSDMD by STING promotes the upregulation of NETs formation in viral pneumonia. **(A)** The expression of STING and GSDMD proteins in neutrophils in the lung tissues of WT and STING-KO mice infected with PR8 on day 5 was visualized using an immunofluorescence assay. The images captured focused on the neutrophil structure along with the released net, indicated by a white arrow (scale bar: 10 μm). MPO is labeled in purple, STING is labeled in green, and GSDMD is labeled in yellow. **(B)** Lung tissue lysates were collected from mice in the Control and PR8 groups and subjected to immunoprecipitation with an anti-STING antibody, an anti-GSDMD antibody, and normal IgG followed by immunoblotting with the indicated antibodies. **(C, D)** Images of immunofluorescence staining for STING and GSDMD in the treated neutrophils in each experimental group were acquired using laser confocal microscopy at a magnification of 400× (scale bar: 10 μm). GSDMD is labeled in green, while STING is labeled in red.

Immunoprecipitation with an anti-STING antibody revealed that STING bound to GSDMD, Cit-H3, and MPO. Similarly, immunoprecipitation with an anti-GSDMD antibody showed that GSDMD bound to STING and MPO (**Figure 8B**). Strong correlations among STING, GSDMD, and NETs-related proteins were found in the lung tissues of mice with PR8-induced viral pneumonia. To investigate the mechanism of action of STING and GSDMD in NETs formation in PR8-induced viral pneumonia, we examined the effects of STING and GSDMD on neutrophils. Lung tissues from WT and STING-KO mice were collected after five days of exposure to PR8 for immunofluorescence analysis. The results showed a decrease in GSDMD expression of the neutrophil after STING knockout (**Figure 8A**). In addition, we found that GSDMD and STING colocalized on neutrophil membranes after PR8 infection of neutrophils isolated from WT mice; however, inhibition of STING reduced GSDMD expression on neutrophil membranes (**Figure 8C**). Neutrophils from WT and STING-KO mice were isolated and infected with PR8. It was observed that GSDMD and STING colocalized on the neutrophil membranes of WT mice, but GSDMD did not aggregate on the neutrophil membranes of STING-KO mice (**Figure 8D**). Therefore, GSDMD-mediated NETs formation in PR8-induced viral pneumonia is dependent on STING.

## Discussion

A multitude of viruses, including H5N1 avian influenza viruses, severe acute respiratory syndrome (SARS) coronaviruses, H1N1 influenza viruses, Middle East respiratory syndrome (MERS) coronaviruses, and H7N9 avian influenza viruses, have induced severe illnesses associated with respiratory transmission and lung inflammation, posing significant global public health concerns. Among the various types of pneumonia caused by influenza viruses, H1N1 pneumonia is the most common, affecting patients at a slightly younger age and leading to a higher mortality rate than pneumonia caused by some influenza viruses (43–45). Hence, models of viral pneumonia induced by the PR8 strain of H1N1 influenza virus are commonly used in animal experiments. Studies have shown that the pathogenesis of inflammation caused by the PR8 influenza virus strain involves a host immune response. In PR8 virus infection, viral hemagglutinin and neuraminidase proteins interact with receptors on host cells, initiating the formation of a complex for viral replication and transcription (44) and thus leading to the release of proinflammatory cytokines such as IL-6, TNF-α and IFNs, which facilitates the recruitment and activation of immune cells in the lungs (45, 46). The successful induction of viral pneumonia is accompanied by pulmonary edema and alveolar structural damage, promoting the extensive infiltration of inflammatory cells and the proliferation of immune cells, constituting crucial factors driving the progression of viral pneumonia (47). Dysregulation of the immune response during PR8 infection can result in excessive inflammation, which can even lead to tissue damage in the liver and gastrointestinal tract (48, 49). Therefore, it is crucial to target host regulatory factors to correct the excessive inflammatory response and maintain optimal immune activity in response to the pathophysiological processes associated with viral pneumonia (50).

STING, a stimulator of interferon gene expression, is the principal signal transducer in the immune system, particularly in the response to viral incursions. STING remains inactive under typical physiological conditions. Activation of STING by the detection of cytoplasmic DNA of pathogen and host origin induces the secretion of type I IFNs and proinflammatory cytokines, which in turn activate host immune responses to clear pathogens, suggesting that the STING signaling pathway plays an important role in the defense against pathogens, such as viruses, bacteria, and fungi (13). Type I IFNs are hallmark cytokines induced via the STING pathway and exhibit both anti-inflammatory and anticancer effects (51). Innate immunity against infections and the subsequent activation of adaptive immunity are both significantly facilitated by type I IFNs. Furthermore, type I IFNs trigger the expression of IFN-stimulated genes (ISGs), whose products efficiently target different phases of pathogen replication and restrict the spread of the pathogen (52). Mechanistic studies indicate that injecting STING agonists directly into tumors can inhibit DNA viral infections and increase the antitumor efficacy of radiotherapy and chemotherapy (53, 54). However, excessive activation of STING leads to sustained elevation of type I IFN levels, resulting in excessive immune responses and triggering inflammatory conditions such as rheumatoid arthritis and pulmonary fibrosis (16, 55). In more serious cases, immune system disorders can be triggered by continuous STING signaling, which leads to the development of autoimmune diseases such as systemic lupus erythematosus and rheumatic diseases (55). Therefore, the activation of STING pathway may elicit either pro-antiviral or pro-inflammatory responses depending on the timing of intervention. Timely inhibition of STING preserves its early antiviral activity while preventing excessive activation–driven inflammation.

In our study, PR8 was found to activate STING signaling and stimulate the immune response. Activation of the STING pathway is crucial for immune responses for viral clearance in viral pneumonia (56). However, overactivation of STING stimulates the overproduction of proinflammatory factors and exacerbates inflammation (54). Twenty-four hours after PR8 influenza virus infection, mice were treated with the STING inhibitor (C-176) in our study. The inhibition of STING reduced the infiltration of inflammatory cells and significantly decreased the levels of inflammatory factors in viral pneumonia. Our findings also revealed that inhibiting STING-dependent NF-κB signaling attenuated lung injury. These findings suggest that C-176 may offer a therapeutic strategy for viral pneumonia. Additionally, no significant difference in viral titers was observed between the PR8 group and the PR8+C-176 group, indicating that C-176 attenuated PR8-induced pulmonary inflammation by inhibiting STING-mediated immune responses rather than directly suppressing viral replication. However, it is important to note that the central role of STING in innate immunity means that its suppression could impair the host’s overall immune response, potentially increasing susceptibility to other infections. Therefore, further research is needed to determine the optimal timing for the intervention of STING pathway.

Our research demonstrated that the PR8 influenza virus triggers the STING pathway, potentially leading to the release of functionally associated proteins produced via immune responses and possibly controlling the proliferation of the related cells. NETs are reticulations that are produced by neutrophils and released into the extracellular space in response to stimuli. NETs function primarily to eradicate pathogenic microorganisms through the release of antimicrobial peptides and serve as a physical barrier to prevent the spread of infection (57). However, NETs also have negative effects. Excessive accumulation of NETs can cause local tissue damage during inflammation. In the presence of a high concentration of neutrophils, NETs tend to form large aggregates, which can accelerate the formation of intravascular thrombi during infections (58). Moreover, cytokine storms, driven by significant increases in the levels of inflammatory cytokines, are the predominant complication of viral infections (59). NETs can increase the inflammatory response; for instance, NETs activate the caspase-1 inflammasome to release active IL-1β, which in turn increases sodium urate crystal-mediated stimulation of NETs release (60–62). Interestingly, accumulating evidence indicates that the extracellular mesh structures known as NETs are produced and released by activated neutrophils, exacerbating inflammation through the STING signaling pathway, contributing to pathological processes such as acute lung injury, cerebral hemorrhage, neuroinflammation, and neuronal death (63–65). Our investigation revealed significant activation of neutrophil degranulation signaling in PR8-induced viral pneumonia. However, treatment with STING inhibitors during the course of PR8-induced viral pneumonia reversed the formation of NETs, suggesting that inhibiting the STING pathway may alleviate viral pneumonia by regulating the formation of NETs. Thus, the STING pathway may play a role in viral pneumonia by regulating the production of inflammatory factors and the formation of NETs. Importantly, we found that STING-KO mice were not susceptible to viral inflammation and that NETs formation was significantly reduced after virus infection in STING-KO mice. Consistently, the absence of STING did not lower the viral titers in the lungs, supporting that the role of STING in viral pneumonia is not associated with viral replication. These results further validate the effectiveness of targeting STING to regulate NETs formation as a treatment strategy for viral pneumonia.

A question arises as to how STING regulates the formation of NETs in viral pneumonia. Our transcriptomic analysis suggested that the GSDMD pathway is a likely pathway through which STING regulates NETs in viral pneumonia. Our CoIP analysis demonstrated that STING bound to GSDMD, PAD4, and MPO, while GSDMD also interacted with NETs-related proteins such as PAD4 and MPO. PAD4, which is expressed predominantly in granulocytes, is recognized as a key driver of NETs formation and is observable within the nucleus of quiescent neutrophils (66). The capacity of PAD4 to citrullinate histones and alter histone-DNA associations implies its involvement in transcriptional control, an aspect corroborated by our findings. In addition, in viral pneumonia, the expression of STING and GSDMD on neutrophils is increased. However, the expression of GSDMD was reduced on the neutrophils of the lungs of virus-infected STING-KO mice. Furthermore, our *in vitro* experiments demonstrated that STING and GSDMD colocalized to the membrane of neutrophils isolated from WT mice after virus stimulation, but that the membrane expression of GSDMD was reduced in neutrophils isolated from STING-KO mice following viral infection. These findings further confirmed that viral infection activates STING to regulate the membrane localization of GSDMD, influencing pore formation in the neutrophil membrane.

GSDMD, a 482 amino acid-residue cell membrane protein, exists in an inactive form in the cytoplasm, where its N-terminal and C-terminal structural domains are connected by a central linker region. Stimulation of the GSDMD protein leads to protease-mediated cleavage within its linker region, generating two functional fragments: the N-terminal domain (GSDMD-N) and the C-terminal domain (67). GSDMD-N plays a pivotal role in the formation of pores within the cellular membrane, leading to perforation of the membrane. These pores function as conduits for the egress of intracellular cytokines and cytoplasmic constituents, thereby triggering apoptosis and eliciting an inflammatory response (67, 68). Notably, cleaved GSDMD-N plays a pivotal role in pore formation in the neutrophil membrane. Furthermore, GSDMD is involved in various diseases, including sepsis and neuroinflammatory conditions, via the STING signaling pathway (69, 70). Combined with our findings, these observations indicate that the pore-forming effect of GSDMD on neutrophil membranes depends on STING signaling, which leads to NETs formation and exacerbates the progression of viral pneumonia. In future studies, we will further investigate the mechanism by which the STING pathway activates GSDMD to promote the formation of NETs.

**Figure 9:**
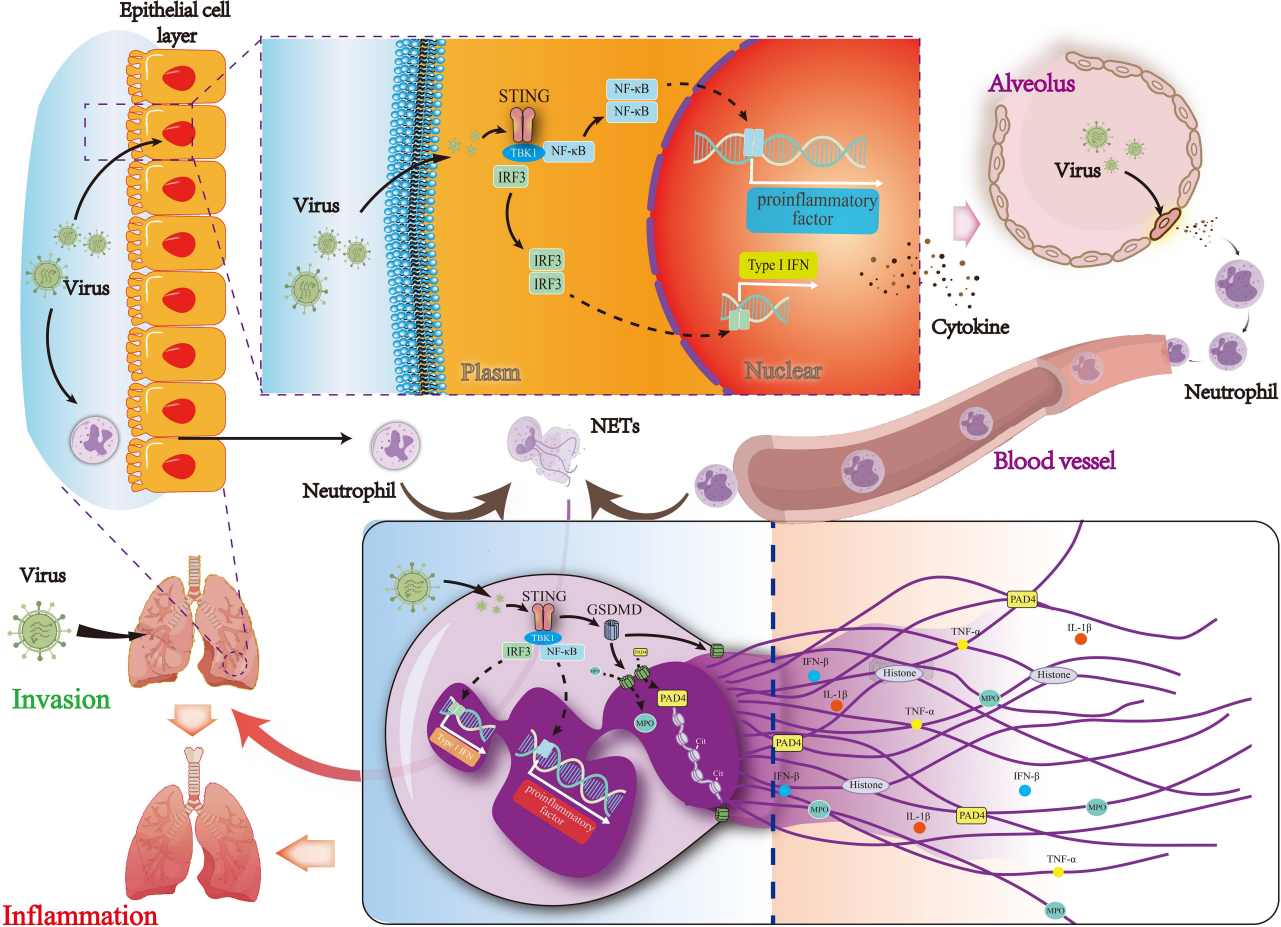
Mechanisms by which STING regulates NETs formation via the activation of GSDMD in viral pneumonia. The STING signaling pathway and its downstream signaling molecules in lung epithelial cells are activated by the virus to produce type I interferon, which leads to the production of pro-inflammatory and chemokine factors and then recruit neutrophils. GSDMD activity is regulated by the activation of STING in neutrophils, leading to the formation of NETs and thereby exacerbating viral pneumonia.

Taken together, the current study assessed the feasibility of targeting STING for the treatment of viral pneumonia and elucidated the role of STING in regulating NETs formation in viral pneumonia. We found that pharmacological STING inhibition reduced lung inflammation in mice with viral pneumonia and decreased virus-induced NETs formation. Moreover, our study revealed that STING-mediated activation of GSDMD induced NETs formation in viral pneumonia, exacerbating this condition (**Figure 9**). In conclusion, targeting STING is a viable therapeutic approach for viral pneumonia, and inhibiting STING also reduces GSDMD activation, thereby decreasing NETs formation and slowing the progression of viral pneumonia.

## Data Availability

The primary data supporting the findings of this study are openly available in the NCBI Sequence Read Archive (SRA) under accession number PRJNA1282064.
