# Supplementary material for: STING regulates NETs formation by activating GSDMD in influenza viral pneumonia
Source: Front Immunol. 2025 Jul 1;16:1598902. doi: 10.3389/fimmu.2025.1598902 (PMC12260538; doi:10.3389/fimmu.2025.1598902)
Supplement: Supplementary file 1 [file DataSheet1.pdf]

Supplementary data

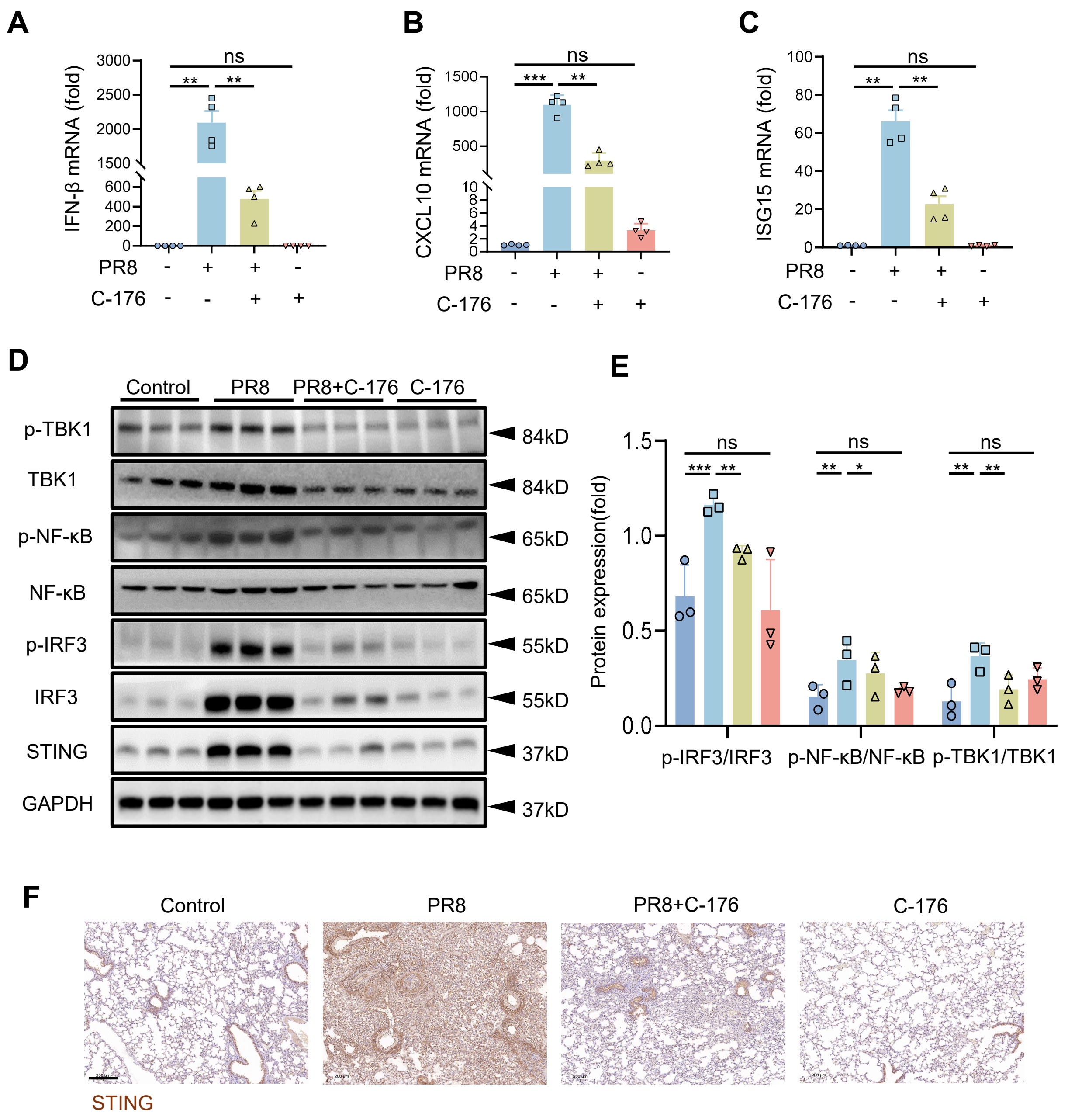

Supplementary Fig.1.A-C:The mRNA levels of IFN-β, CXCL-10 and ISG15 were detected by RT-qPCR (n=4). D:Expression levels of TBK1, NF-κB, IRF3, STING and their phosphorylation were determined by Western blot and quantified using GAPDH as a standard(n=3). E:Protein quantification was analysed by Image J according to the results of D. F:Immunohistochemistry of STING (scale bar =200um). Data are presented as the mean ± SD, *p < 0.05, **p < 0.01, and ***p < 0.001, ****p < 0.0001, and ns indicating no significant difference between the two groups.

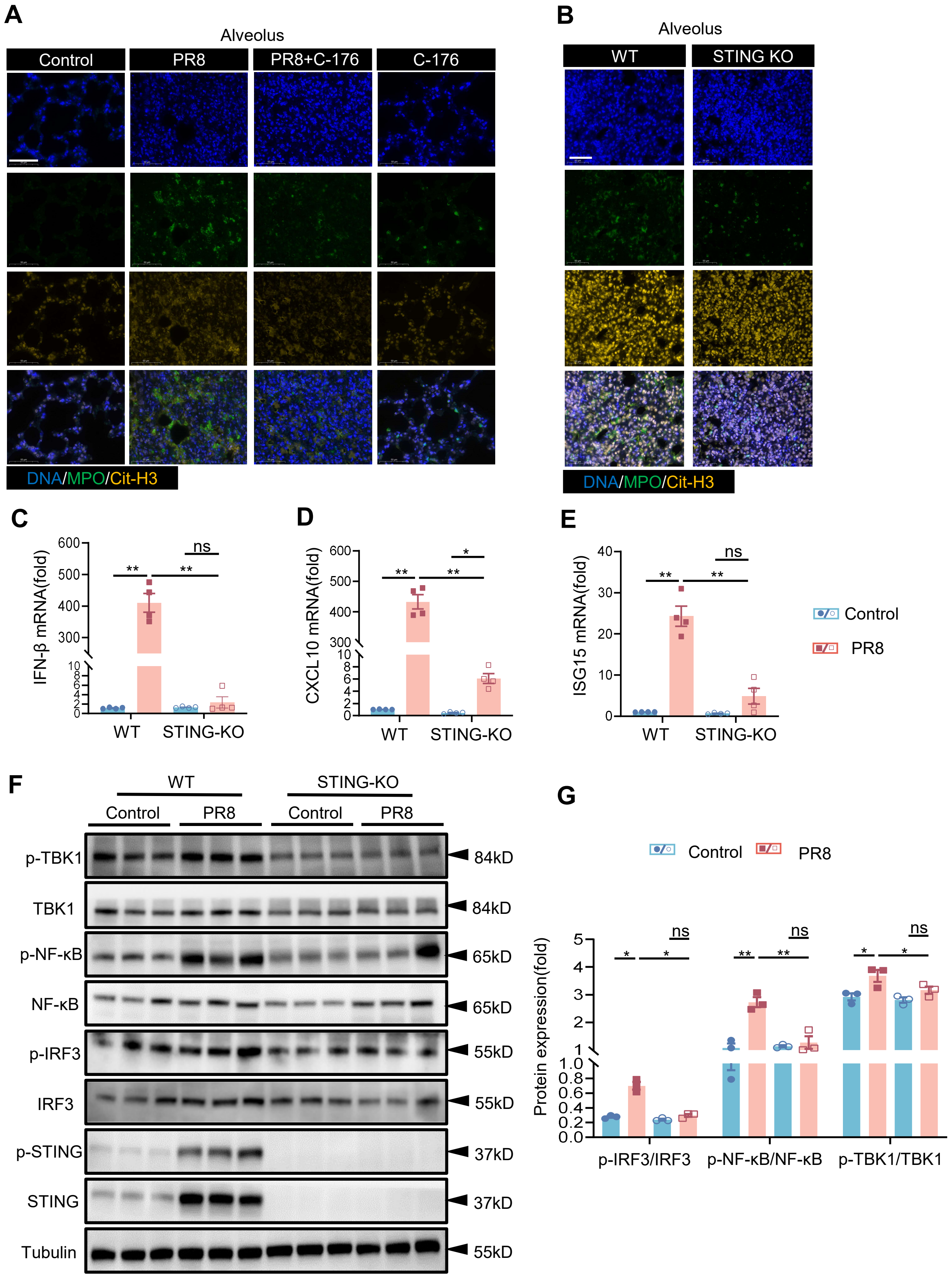

Supplementary Fig. 2.A-B: The formation of NETs in the alveoli of mice in each group was detected by immunofluorescence (scale bar =50um). C-E: The mRNA quantification of IFN-β, CXCL-10, and ISG15 was conducted via RT-qPCR(n=4).F: The levels of TBK1, NF-κB, IRF3, STING, and their respective phosphorylation states were assessed through Western Blot with Tubulin serving as a standard for quantification(n=3). G: Protein quantification was carried out as per the methodologies outlined in F's findings utilizing Image J software. Statistical significance denoted as * $p < 0.05$, ** $p < 0.01$, *** $p < 0.001$, **** $p < 0.0001$, ns indicating no significant difference between the two groups.

Supplementary Table 1. Gene Primers

Gene name	Gene code	Primers
interleukin 6	IL-6	Forword: TGGTCTTCTGGAGTACCATAGC
		Reverse: TGTGACTCCAGCTTATCTCTTGG
interleukin-1 beta	IL-1β	Forword: TGCCACCTTTTGACAGTGATG
		Reverse: TGATGTGCTGCTGCGAGATT
tumor Necrosis Factor	TNF-α	Forword: TGCGGAGGCCTAAAGTCTCT
		Reverse: AGGGATGTTGGCTGCCTTTT
Interferon beta	IFN-β	Forword: TGGGAGATGTCCTCAACTGC
		Reverse: CCAGGCGTAGCTGTTGTACT
ISG15 Ubiquitin Like Modifier	ISG15	Forword: GGGGCCACAGCAACATCTAT
		Reverse: AGCCAGAACTGGTCTTCGTG
C-X-C motif chemokine ligand 10	CXCL-10	Forword: CCAAGTGCTGCCGTCATTTT
		Reverse: AGCTTCCCTATGGCCCTCAT
glyceraldehyde-3-phosphate dehydrogenase	GAPDH	Forword: CCCTTAAGAGGGATGCTGCC
		Reverse: TACGGCCAAATCCGTTCACA
